# Complete Genome Sequence of *Turicibacter* sp. Strain H121, Isolated from the Feces of a Contaminated Germ-Free Mouse

**DOI:** 10.1128/genomeA.00114-16

**Published:** 2016-03-24

**Authors:** T. A. Auchtung, M. E. Holder, J. R. Gesell, N. J. Ajami, R. T. D. Duarte, K. Itoh, R. R. Caspi, J. F. Petrosino, R. Horai, C. R. Zárate-Bladés

**Affiliations:** a Alkek Center for Metagenomics and Microbiome Research and Department of Molecular Virology and Microbiology, Baylor College of Medicine, Houston, Texas, USA; b Laboratory of Molecular Ecology and Extremophiles, Department of Microbiology, Immunology and Parasitology, Federal University of Santa Catarina, Florianopolis, Brazil; c Bio-Technical Center, Japan SLC, Inc., Hamamatsu, Shizuoka, Japan; d Laboratory of Immunology, National Eye Institute, National Institutes of Health, Bethesda, Maryland, USA; e Laboratory of Immunoregulation, Department of Microbiology, Immunology and Parasitology, Federal University of Santa Catarina, Florianopolis, Brazil

## Abstract

*Turicibacter* bacteria are commonly detected in the gastrointestinal tracts and feces of humans and animals, but their phylogeny, ecological role, and pathogenic potential remain unclear. We present here the first complete genome sequence of *Turicibacter* sp. strain H121, which was isolated from the feces of a mouse line contaminated following germ-free derivation.

## GENOME ANNOUNCEMENT

Members of the bacterial genus *Turicibacter* have been cultured or detected by PCR analysis, primarily from a variety of animal hosts or their waste (e.g., reference 1). However, only two isolates have been described thus far: the type strain *Turicibacter sanguinis* MOL361 (2), and the facultatively anaerobic methanol wastewater strain ZCY83 (3). Both isolates were observed to be Gram-positive non-spore-forming irregularly shaped rods. The phylogenetic position of *Turicibacter* has been uncertain; the 16S rRNA gene sequence of MOL361 might place it within the phylum *Firmicutes*, but not deeper with any degree of confidence (2). Later analyses placed *Turicibacter* in the class *Bacilli* (4), and then tentatively in the class *Erysipelotrichia* (5). The draft genomes of two undescribed *Turicibacter* strains were previously sequenced, those of PC909 (6) and HGF1 (GenBank accession no. NZ_AEXQ00000000.1).

*Turicibacter* sp. strain H121 was isolated following a germ-free derivation attempt of an R161H-TCR transgenic mouse line (7) at Taconic Farms (Germantown, NY). Two weeks after germ-free pups were born, contamination by a Gram-positive microbe was detected. Fecal samples from these pups were frozen until reconstituted in germ-free C57BL/6 mice to enrich for viable bacteria. From the feces of these mice, strain H121 was isolated anaerobically on Eggerth-Gagnon plates. The strain also grew on brain heart infusion (BHI) (Fluka) plates at 37°C under anaerobic, but not aerobic, conditions. However, inoculation into BHI, thioglycolate, or chopped meat carbohydrate broths resulted in no detectable growth after 4 weeks under anaerobic conditions at 37°C. Unlike strain MOL361, H121 survived freezing at -80°C in BHI or thioglycolate broth plus 15% glycerol but only survived lyophilization when in 10% skim milk.

High-molecular-weight DNA was extracted from colonies that were grown for 1 week on BHI plates, precipitated with sodium chloride and ethanol (8), and sequenced using the Pacific Biosciences RS II platform, according to the manufacturer’s protocols. Reads were fed into the Celera assembler and annotated using the NCBI tools Glimmer and GeneMark (9, 10).

The complete circular genome sequence of strain H121 comprised 2,622,031 bases, with 34.8% GC content and an average reference coverage of 74. The postfilter read *N*_50_ is 15,432 bases, with a quality value of 0.84. Annotation features include 2,486 genes, 94 pseudogenes, 12 16S rRNAs, 11 23S rRNAs, 13 5S rRNAs, 140 tRNAs, and 60 sporulation genes but no genes for flagella or pili. Of the 12 16S rRNA gene copies, 10 copies were identical across 1,509 nucleotides (nt), and the other 2 copies had a single-nucleotide insertion or substitution. The 10-copy 16S rRNA gene sequence was 99.2% identical to MOL361 and PC909, and 97.7% to ZCY83 (all single copies reported). There is no 16S rRNA gene among the HGF1 draft genome sequence. Phylogeny was inferred using an alignment of select amino acids from up to 400 genes with a database of 3,771 other organisms (11). *Turicibacter* sp. H121 was placed adjacent to the clade containing PC909 and HGF1, and together, they grouped with the halophilic anaerobe *Haloplasma contractile* at the base of the class *Bacilli* (12).

### Nucleotide sequence accession number. 

The sequence of *Turicibacter* sp. H121 has been deposited in NCBI GenBank accession no. CP013476.
